# X-ray beam quality after a mirror reflection: Experimental and simulated results for a toroidal mirror in a 4 ^th^ generation storage-ring beamline

**DOI:** 10.12688/openreseurope.16211.1

**Published:** 2023-10-12

**Authors:** Juan Reyes-Herrera, Rafael Celestre, Marco Cammarata, Raymond Barrett, Matteo Levantino, Manuel Sanchez del Rio

**Affiliations:** 1 European Synchrotron Radiation Facility, Grenoble, 38043, France

**Keywords:** x-ray optics, x-ray mirror, syncrotron radiation, partial coherence

## Abstract

**Background:**

The surface errors found in X-ray mirrors constitute a limiting factor for preserving beam quality. This is particularly important when the X-ray beam has low emittance and a significant coherence fraction, like in newly upgraded synchrotron storage rings.

**Methods:**

We studied the fringes observed in the image of an undulator-produced X-ray beam reflected by a high-quality toroidal mirror. The measurements and simulations were performed using different conditions: a photon beam either monochromatic or with large bandwidth, reflected by a mirror with variable curvature.

**Results:**

The experimental data are compared with up-to-date simulation including partial coherence.

**Conclusions:**

The observed fringes in the unfocused beam correlate with low spatial frequency structures in mirror profiles, irrespective of beam coherence. Both classical ray tracing and partially coherent simulations through coherent mode decomposition are confirmed as accurate methods for such simulations.

## Plain language summary

In this study, researchers focused on the surface errors found in X-ray mirrors and their impact on beam quality. These errors can be problematic, especially when dealing with X-ray beams coming from low emittance (a measure of beam size and divergence) electron beam sources and a significant coherence fraction (indicating the level of wavefront coherence).

The researchers specifically investigated the fringes observed in the image of an X-ray beam produced by an undulator and reflected by a high-quality toroidal mirror. They conducted measurements and simulations under different conditions, such as using a monochromatic photon beam or one with a wide range of wavelengths, and varying the curvature of the mirror.

## Introduction

Optical instruments in 4 ^*th*^ generation storage-rings share a common need: they require perfect optical surfaces ^1^. The X-ray optical systems at the beamlines are more demanding than those using other wavelengths. A reason is a need to work in grazing incidence with reflective optics. The irregularities in the optical surface enhance the beam distortion. X-ray optics must i) be resistant to deformations originated by thermal load, ii) have a surface shape that follows closely ideal mathematical surfaces that produced perfect focusing ( *e.g.* ellipsoids, paraboloids, *etc.*), and iii) have a perfect surface finish. Moreover, the optics must be stable to vibrations and thermal drifts, and resistant to degradation and contamination for years. For example, more details regarding these requirements can be found in 1.

The quality of the mirrors has improved a lot adapting to the evolution of beam quality. Typical values 25 years ago of 1-4 *µ*rad rms (root-mean-square) slope error and 1–4 nm rms microroughness (see, *e.g.* 2) have improved to sub-0.5 *µ*rad or even better around the turn of the century. In the last years, the mirrors and other optics reached sub-100 nrad slope errors and 1–2 nm shape errors, something almost unthinkable a few decades ago ^1^. In the past, with large incoherent beams, the critical factor was the slope errors that enlarge the focused beam cross-section (with also a consequent decrement in beam intensity). The reduction of the intensity peak caused by mirror microroughness was usually small in super-polished mirrors. Under these conditions, the enlargement of the focused beam due to slope errors was typically studied with ray tracing, using measured ^3^ or simulated ^4^ mirror profiles. With low-emittance beams, but always incoherent, it was possible to detect fine structures in the focused beam (see *e.g.* 5), related to the low-spatial frequencies of the surface shape. For the accurate simulation of these features, it is necessary to know the real mirror profile that is typically obtained from metrology measurements. The improvement of the beam emittance in storage rings increased the coherence fraction of the X-ray beams, boosting the experiments exploiting beam coherence and popularizing the imaging measurements. It was found that the beams were contaminated with structures due to diffraction (typically due to surface errors and limited accepted numerical aperture). Moreover, the need to use beams out of focus made these and other effects more evident. When the coherent fraction is high, the X-ray beam can be treated as a single wavefront, and typical results like the Marechal criterium (condition to obtain a Strehl ratio equal to 0.8) were used to define mirror quality for focused beams ^1^. But, as soon as there is the need of working out of focus the Strehl ratio needed to preserve coherence must increase to 0.97 or even better ^1^.

For hard X-rays produced in 4 ^*th*^ generation storage rings the coherence fraction is a few percent, therefore the criteria developed for fully coherent beams are not applicable ^6^. It was found that the ray tracing simulations overestimated the effect of the slope errors, therefore new hybrid methods were proposed ^6^. These hybrid methods fail to reproduce fringes and structures found when imaging the beam out-of-focus. This is because the effects that are due to coherence are introduced *via* a convolution of the diffraction angular pattern with the beam divergence, therefore the local effects of the mirror irregularities are lost. More sophisticated partial-coherence studies can be done to include all these effects, like wave optics and coherent mode decomposition of the undulator source ^7, 8^.

We study here the degradation of the beam quality by the surface errors in a toroidal mirror installed at the ID09 beamline at the new Extremely Brilliant Source (EBS) of the European Synchrotron Radiation Facility (ESRF) a 4 ^*th*^ generation storage ring. Experimental and simulated data are compared. The observed diffraction fringes are originated from the height errors of the mirror surface. This has been checked numerically using ray tracing and partial coherence simulations based on coherent mode decomposition and wave optics propagation. The simulations used the measured mirror profile recorded at the ESRF Metrology laboratory. It is also shown that simple experimental imaging setups, such as the one used here, can be used to obtain an approximated mirror profile.

## Methods

### Experimental

**Figure 1.**
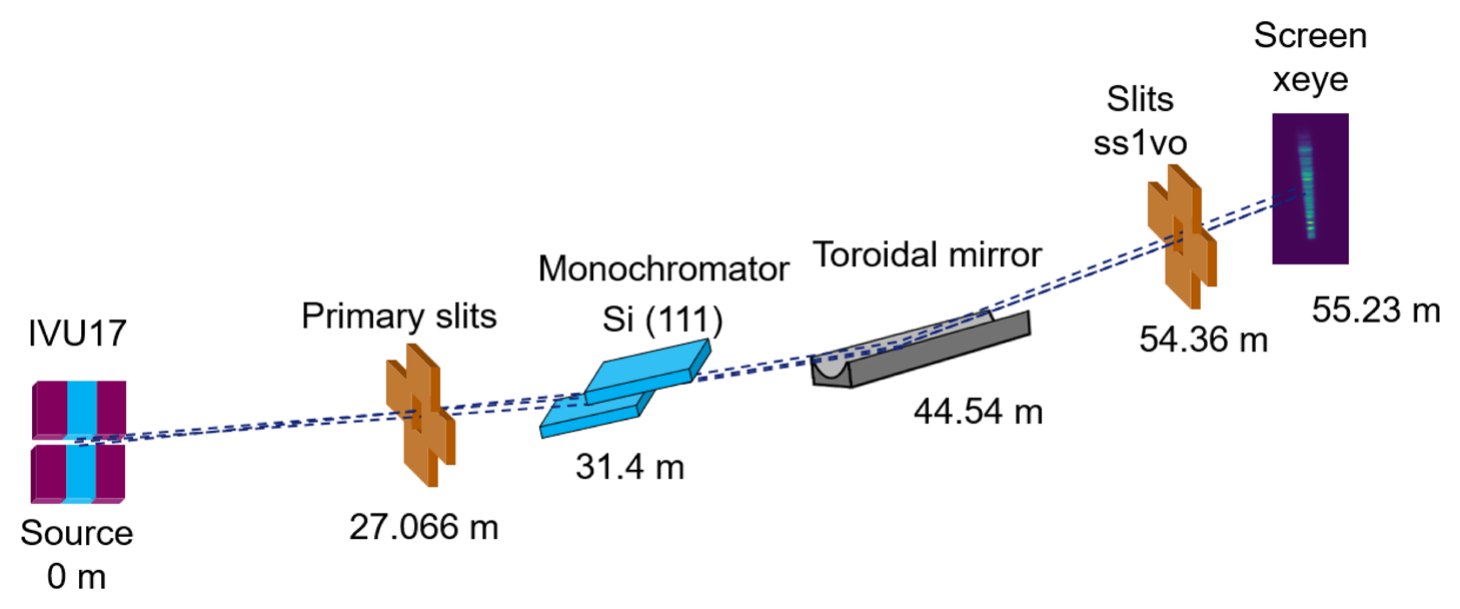
Experimental setup at the ID09 beamline, including the position of the elements with respect to the source.

The experimental data have been collected at the ID09 ESRF beamline at the EBS-ESRF storage ring ^1^, using an IVU17 undulator set to have its first harmonic at an energy of E = 18.5 keV (deflection parameter K = 0.417). Downstream from the source we find the entrance slit at 27.066 m, a toroidal mirror at 44.54 m (grazing angle of 2.5 mrad). The cross section intensity profiles of the photon beam were acquired by a fluorescent screen at 55.23 m ( *xeye*). Relevant ID09 beamline optical components for this study are shown in Figure 1.

The toroidal mirror was supplied by SESO ^TM^ and installed in the beamline in 2017, replacing the one that was working since 2001 ^9^. The mirror has a clear aperture of 600 mm × 10 mm, it is made of Si with a Pd coating (500 ± 100Å), it is mounted on a mechanical bender (designed by SESO ^TM^) to have adjustable meridional radius (along the X-ray beam), its sagittal radius (perpendicular to the beam) is 46.34±0.1 mm. The profile used for the simulations in this work has been measured at the ESRF Metrology laboratory. The mirror has 0.5 *µ*rad rms slope error, 18 nm rms of height error and a micro-roughness of 2.8 Å. Measurements were done to calibrate the *xeye* and obtain the accurate pixel size (see following subsection). A data set with all results has been deposited in Zenodo data repository ^10^. Experimental log can be found in the project Zenodo repository ^11^. Some measurements were dedicated to recording the energy spectrum around the energy resonance ^2^ (see subsection Sprectal measurements).

Images of the photon beam cross section at the *xeye* monitor were acquired in different experimental configurations (runs):

1. **Beam profile *vs* photon energy,** with primary slits 1 mm × 1 mm, meridional radius of mirror at 20 km, energy scan from 16 keV to 19 keV to cover all the resonance peak (see Spectral measurements subsection) with 30 eV step.
2. **Beam profile *vs* photon energy with narrow H slit,** with primary slits 0.1 mm (horizontal) × 1 mm, toroidal mirror meridional radius of 20 km, energy scan from 16 keV to 19 keV with 30 eV step.
3. **Beam profile *vs* curvature radius (monochromatic beam),** with primary slits at 1 mm × 1 mm, photon energy at 18.5 keV, toroidal mirror meridional radius scanned from 20 km to 5 km.
4. **Beam profile *vs* curvature radius (pink beam),** with primary slits at 1 mm × 1 mm, photon white beam, toroidal mirror meridional radius scanned from 20 km to 5 km.
5. **Beam profile *vs* vertical scan of the slits *ss1vo*,** with primary slits at 1 mm × 1 mm, monochromatic beam at 18.5 keV, toroidal mirror meridional radius of 20 km, slit *ss1vo* gap of 10 *µ*m in vertical and scanned in the vertical axis from -0.3 mm to 0.6 mm, using 361 steps.

### Simulations

The ray tracing method is a powerful technique to predict the performance of an optical system. For synchrotron radiation applications, the popular code SHADOW ^12^ (version 23.1.4) is used in the OASYS environment ^13^ (version 1.2.130). In this ray tracing code, a ray is a mathematical entity fully specified by four vectors and two phases: the starting position **r**, the direction **v**, the electric fields **E**_*π*_ and **E**_*σ*_ ( *σ* and *π* refer to the parallel and perpendicular polarizations) and their phases *ϕ _π_* and *ϕ _σ_*). The source is created using the Monte-Carlo method to sample rays with the spatial, angular and energy distribution of the synchrotron sources. Each ray of the source represents a small pencil photon beam that is traced through an optical system consisting of a number of optical elements (mirrors, gratings, crystals, *etc.*). It is therefore suitable for simulating incoherent beams, because the beam intensity is computed as the (incoherent) sum of the intensity of the different rays. Values like beam cross sections, energy resolution, *etc.* are calculated by post-processing (histogramming, integration, visualization, *etc.*). Although ray tracing is originally restricted to simulate incoherent beams, some correction algorithms to include diffraction effects that happen with coherent radiation were developed ^6^ and included in the OASYS suite.

Other methodologies, based on physical optics, exploit the well-known propagation of coherent wavefronts. If the source is fully coherent, a single wavefront is sufficient to describe the photon beam. Wavefront propagation techniques can be used to transport the wavefront along the beamline. Radiation from free electron lasers is fully coherent in good approximation, and wavefront propagation can be used directly. On the contrary, the coherence of the radiation produced by new storage rings depends on the wavelength. The coherence fraction is high at large wavelengths (soft X-rays) and decreases quickly for hard X-rays. In that case, the partial coherence is treated by propagating a set of wavefronts that all together describe the undulator radiation. The set of wavefronts used in this work are calculated using coherent mode decomposition. The wavefronts are assigned to coherent modes, which are the eigenfunctions of the cross-spectral density, and can be numerically calculated for the undulator source. A full treatment of the coherent mode decomposition with two-dimensional wavefronts was implemented in the COMSYL package ^7^. Based on that, a new light method was recently proposed ^8^, and it was demonstrated to be very efficient for simulations at hard X-rays. This method, as implemented in the WOFRY1D addon in the OASYS package, is used here. We have used experimental metrology data to account for the simulations for the effect of mirror errors. OASYS work-spaces for ray-tracing and wavefront simulations and Python scripts for the data analysis are included in a Zenodo repository ^11^.

### Calibration of pixel size

**Figure 2.**
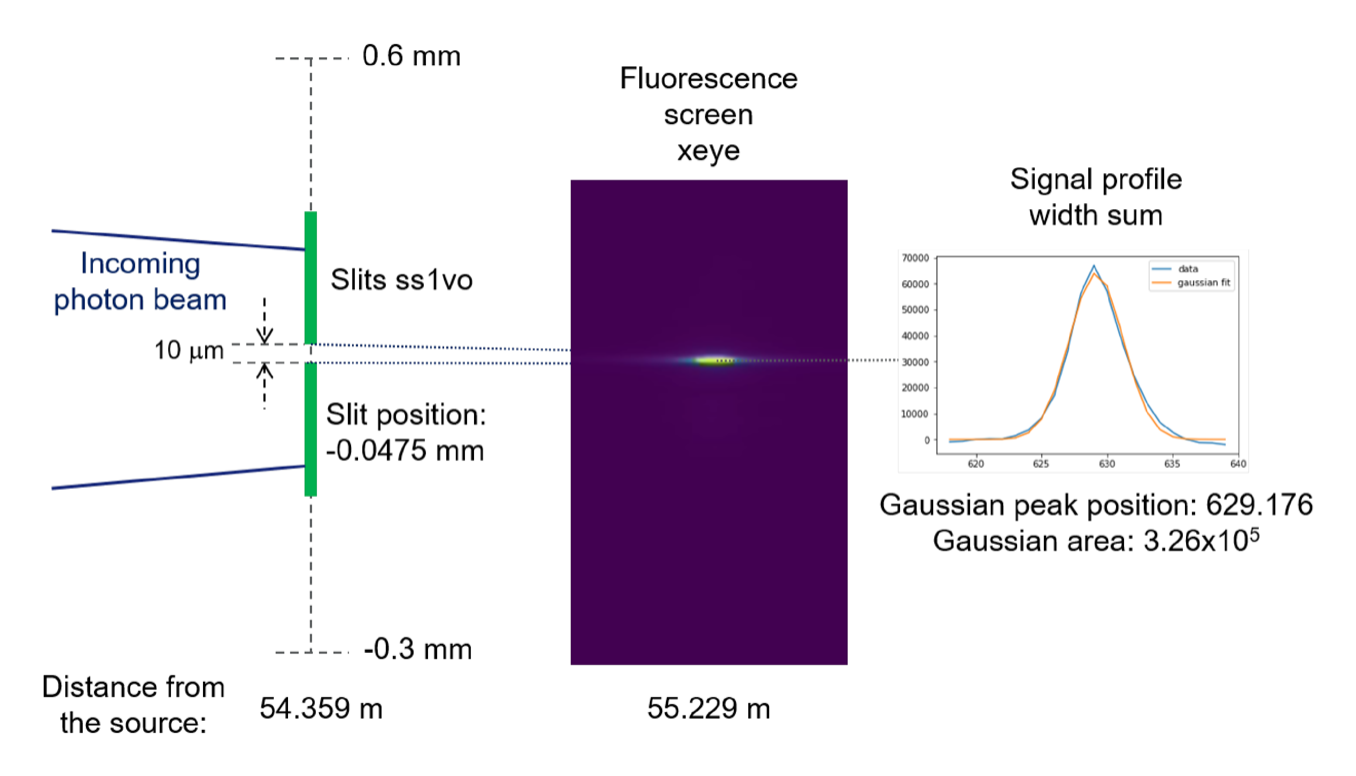
Scheme to show how we obtain the signal intensity (Gaussian area) as a function of the *ss1vo* vertical position, for example for the position -0.0475 mm.

Determining the *xeye* pixel size is a necessary step for displaying correctly the measured intensity profiles. We used only measured data from run 5. On one hand, we can obtain the 1D vertical profile in the function of the slit position by getting the signal intensity for each *ss1vo* position, to carry out this task we fitted a Gaussian curve to the sum in width of the signal for each slit position step, *e.g.,* schematically shown Figure 2.

**Figure 3.**
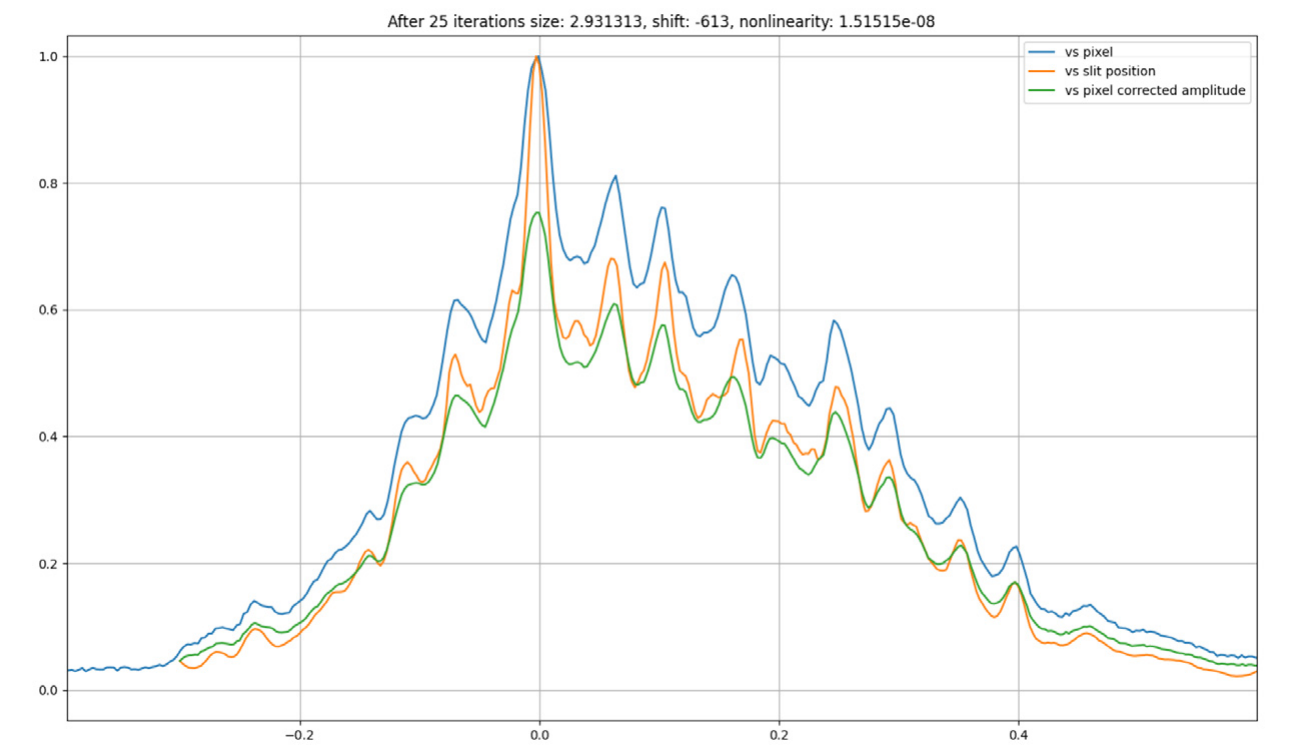
Best matching between 1D profiles as a function of the *ss1vo* and the pixel position in the *xeye* beam profile monitor, pixel size respect to the slits is 2.93 *µ*m.

On the other hand, we summed all the images recorded while scanning the slit, and then obtain the 1D vertical profile by integrating the intensities along the width axis. Now, we can obtain the pixel size with respect to the *ss1vo* by matching both 1D profiles. Since the horizontal axis units from each 1D profile are completely different (mm and pixels, respectively) it is necessary to optimize not only a pixel size but a shift as well. A Python routine ^3^ was used to perform an optimization of both parameters. Best pixel size respect to the *ss1vo* is 2.93 *µ*m, Figure 3.

**Figure 4.**
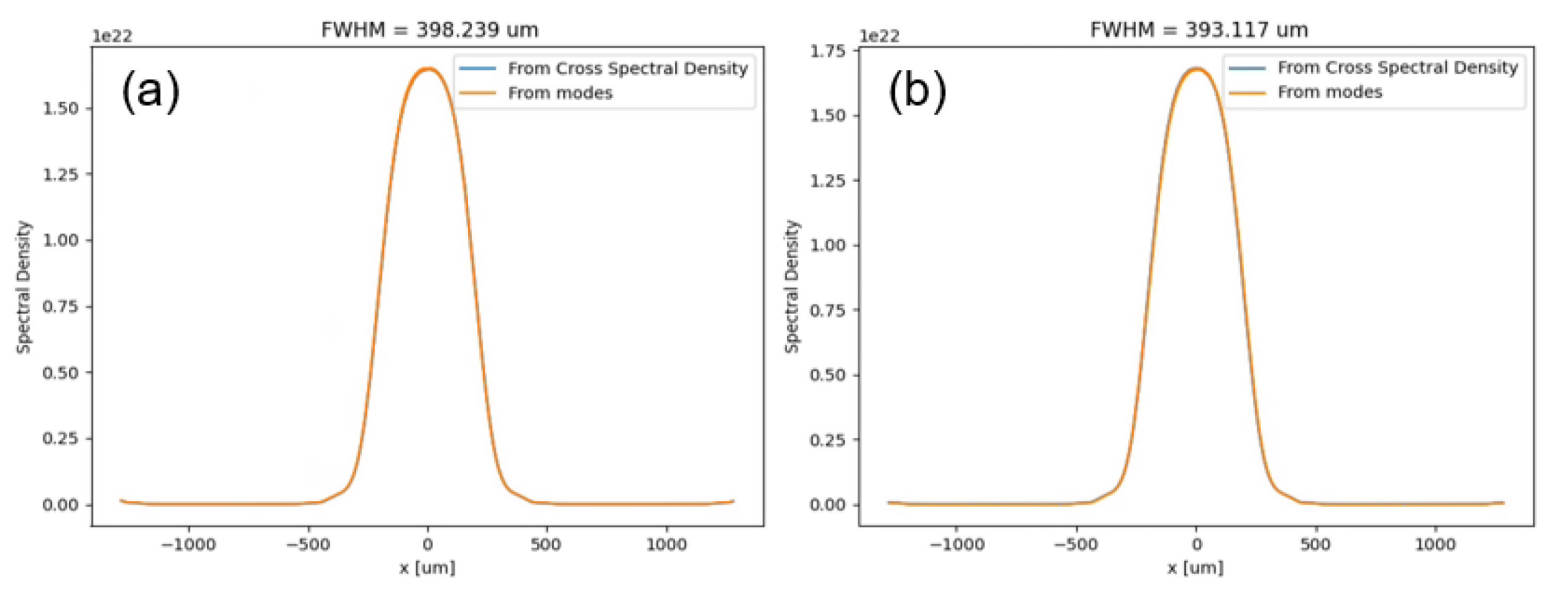
Simulation using wave optics (Wofry1D) of the beam size at the *sss1vo* ( **a**) and *xeye* ( **b**) positions corresponding to 18.5 keV and 20 km of meridional toroidal mirror radius (using perfect surfaces for simplicity).

As explained above, the found pixel size is in respect to the *ss1vo*, in order to project it to the proper *xeye* position, we can consider the vertical focusing of the photon beam due the toroidal mirror. Using ray tracing, SHADOW ^12^, and 1D wavefront propagation, WOFRY 1D ^8^ (version 1.0.31) see Figure 4, to observe the beam size ratio reduction between the *ss1vo* and the *xeye* screen positions ( Figure 1), we determined a *xeye* pixel size of 2.87 *±* 0.02 *µ*m.

### Spectral measurements

**Figure 5.**
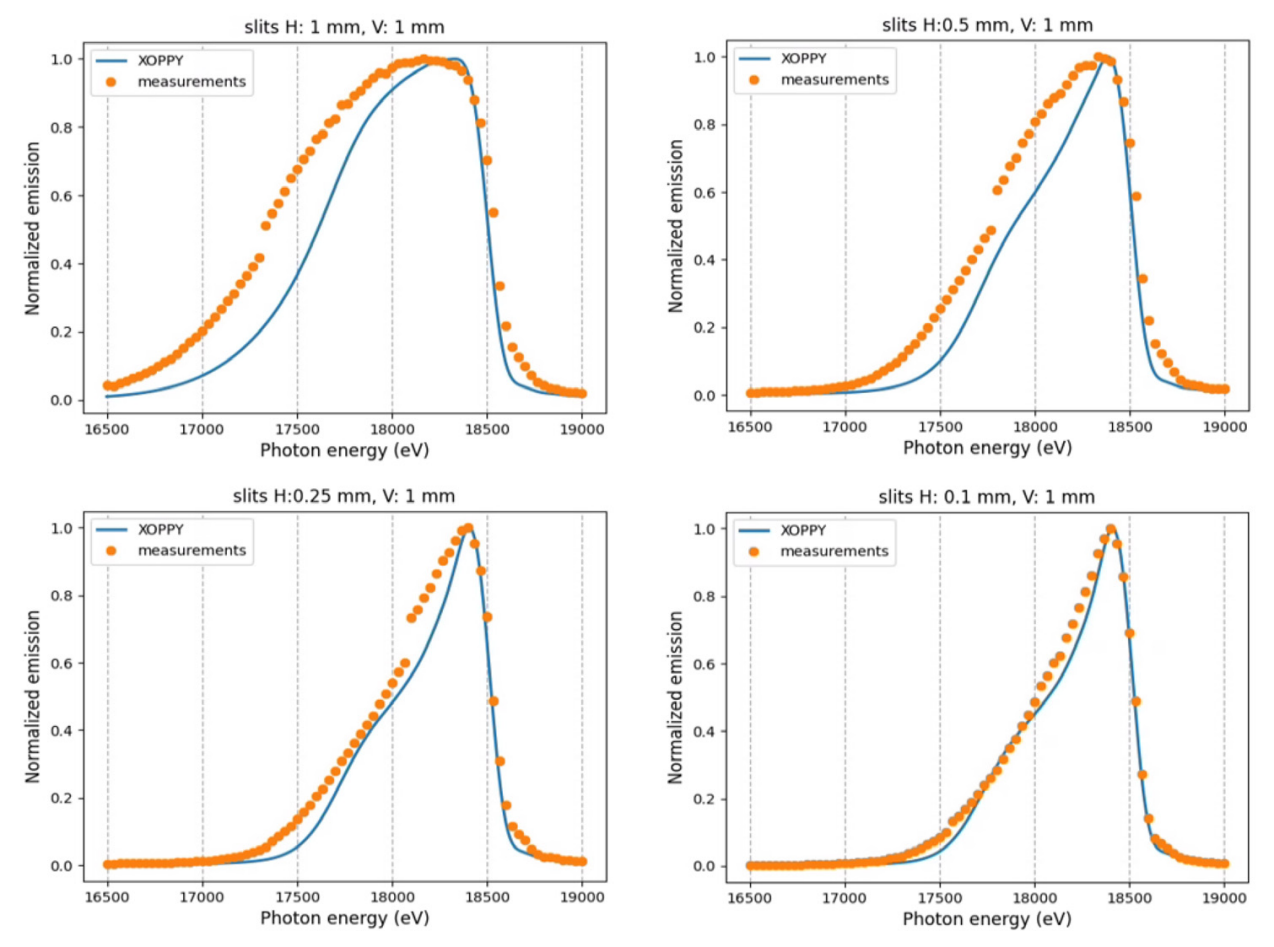
Photon beam spectra from different horizontal primarily slit apertures (slits H). The measurements are compared with theoretical spectra calculated using XOPPY.

In order to study the effect of the horizontal primarily slit, we measured the beam intensity as a function of its energy for different horizontal apertures. We fixed the undulator gap at 10.095 mm and scanned the photon energy using the monochromator. Measurements are compared with XOPPY ^13^ (version 1.2.10) simulations in Figure 5.

## Results

### Run 1

**Figure 6.**
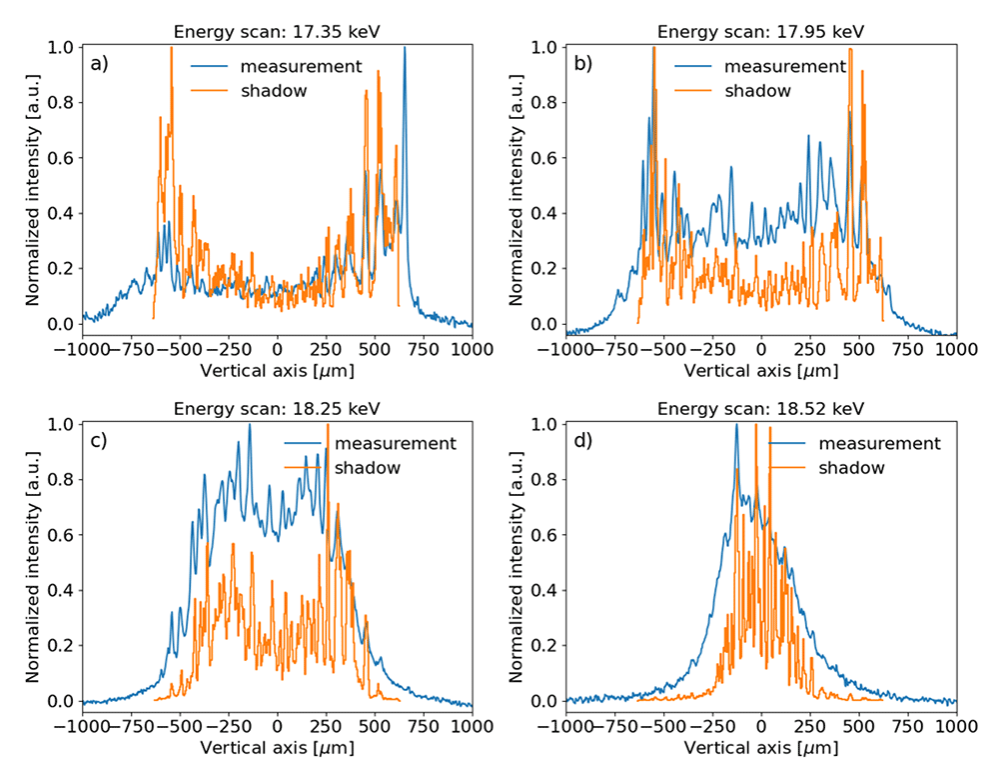
Experimental beam intensity projected in the vertical direction and corresponding ray tracing simulations for several photon energies: **a**) 17.35 keV, **b**) 17.95 keV, **c**) 18.25 keV and **d**) 18.5 keV (resonance). The measured data and simulations were normalized to the peak value. A vertical offset was applied to the experimental data for better comparison.

We recorded 101 images and extracted the vertical beam profiles for different photon beam energies. Some experimental profiles are shown in Figure 6, together with ray tracing simulations. It is noticed how the beam geometry changes as a function of the energy. At the exact resonance (18.5 keV) the image of the well-defined central cone is obtained ( Figure 6d). At lower energies ( Figure 6a–c), the peaks of the ring are visible, getting closer and closer to merge in the central peak. The ray tracing simulations reproduce the trend shown in the measurements.

### Run 2

**Figure 7.**
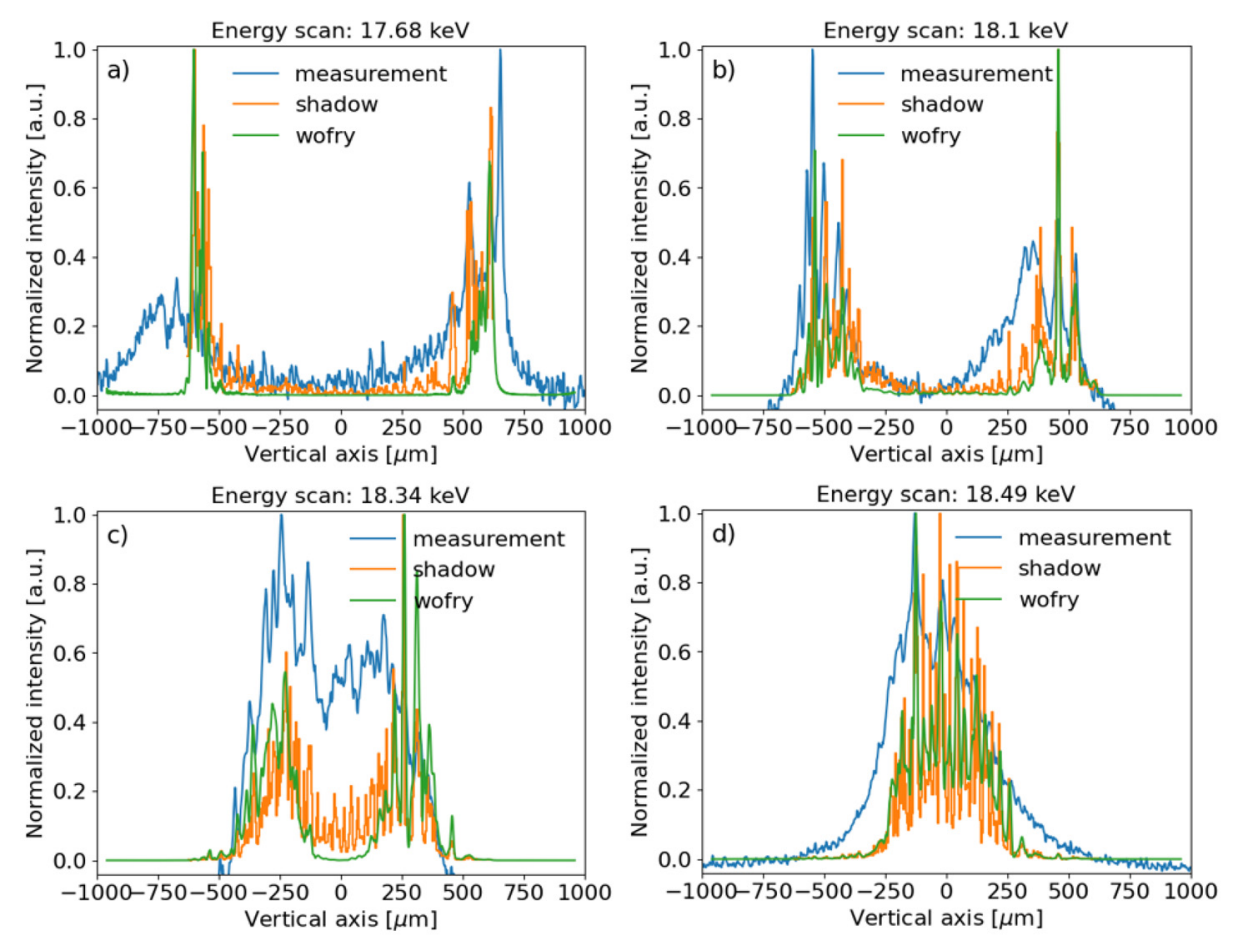
Experimental beam intensity projected in the vertical direction compared to SHADOW (ray tracing) and WOFRY1D (partial coherence) simulations, for several photon energies: **a**) 17.68 keV, **b**) 18.1 keV, **c**) 18.34 keV and **d**) 18.5 keV (resonance). The measured data and simulations were normalized to the peak value. A vertical offset was applied to the experimental data for better comparison.

In this run, the primary slits were closed in the horizontal direction to 0.1 mm, thus reducing the contribution of the sagittal features of the toroidal mirror to the intensity profile of the beam. The results are shown in Figure 7, also including ray tracing (SHADOW) and wavefront simulations using coherent mode decomposition (WOFRY1D).

### Run 3

**Figure 8.**
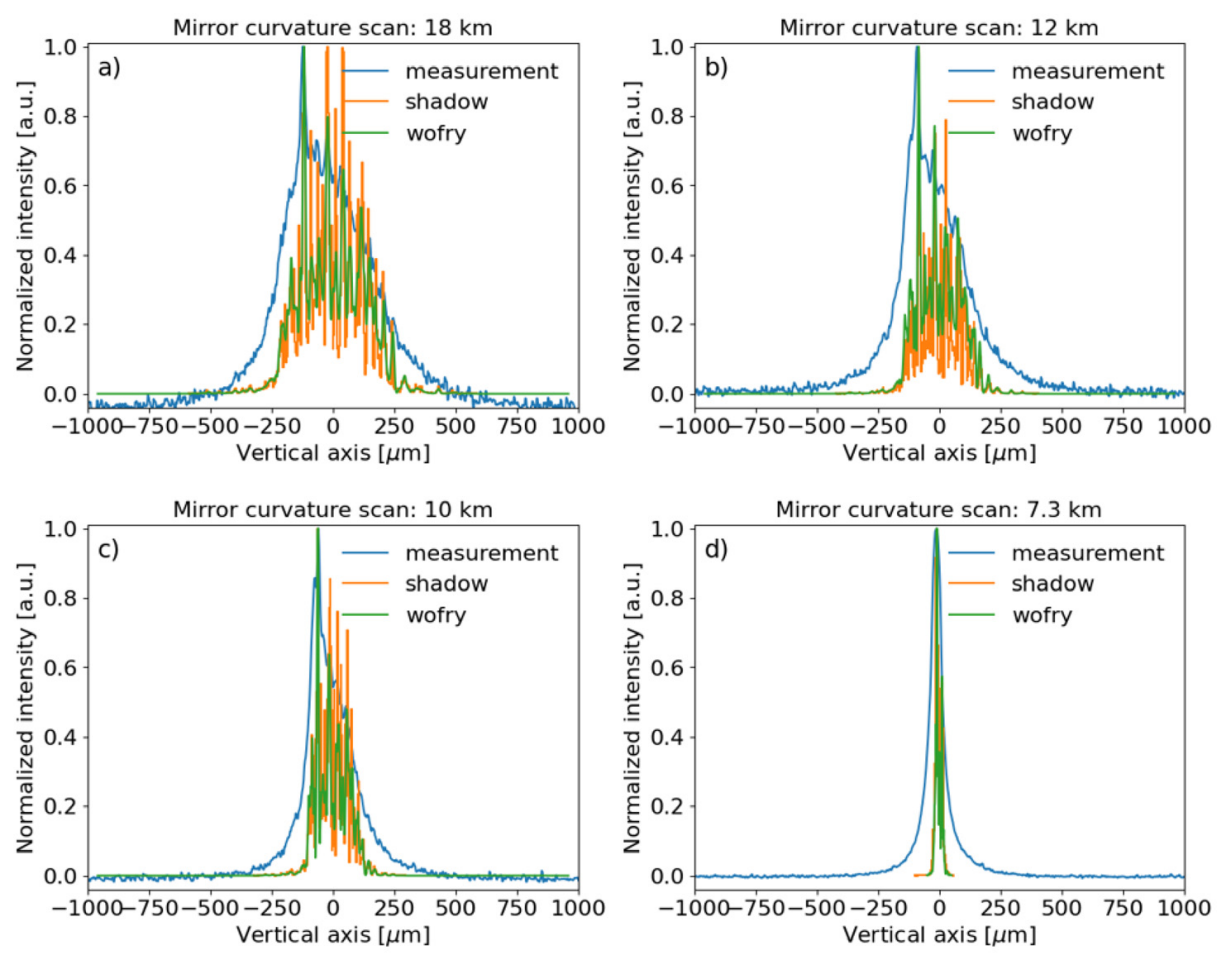
Experimental beam intensity, obtained in monochromatic mode (at the resonance energy), projected in the vertical direction compared to SHADOW and WOFRY1D simulations for different meridional curvature radii of the toroidal mirror: **a**) 18 km, **b**) 12 km, **c**) 10 km and **d**) 7.3 km (minimum measured beam size). The measured data and simulations were normalized to the peak value. A vertical offset was applied to the experimental data for better comparison.

We scanned the curvature radius of the toroidal mirror in monochromatic mode. A coarse scan from 20 km to 5 km with steps of 1 km, for each step 3 images were saved with different exposure times: 1 ms, 3 ms and 10 ms. Then, a fine scan, from 10 km to 5 km with 0.05 km steps, 2 images were recorded for each step with 1 ms and 3 ms exposure times. Some results are shown in Figure 8.

### Run 4

**Figure 9.**
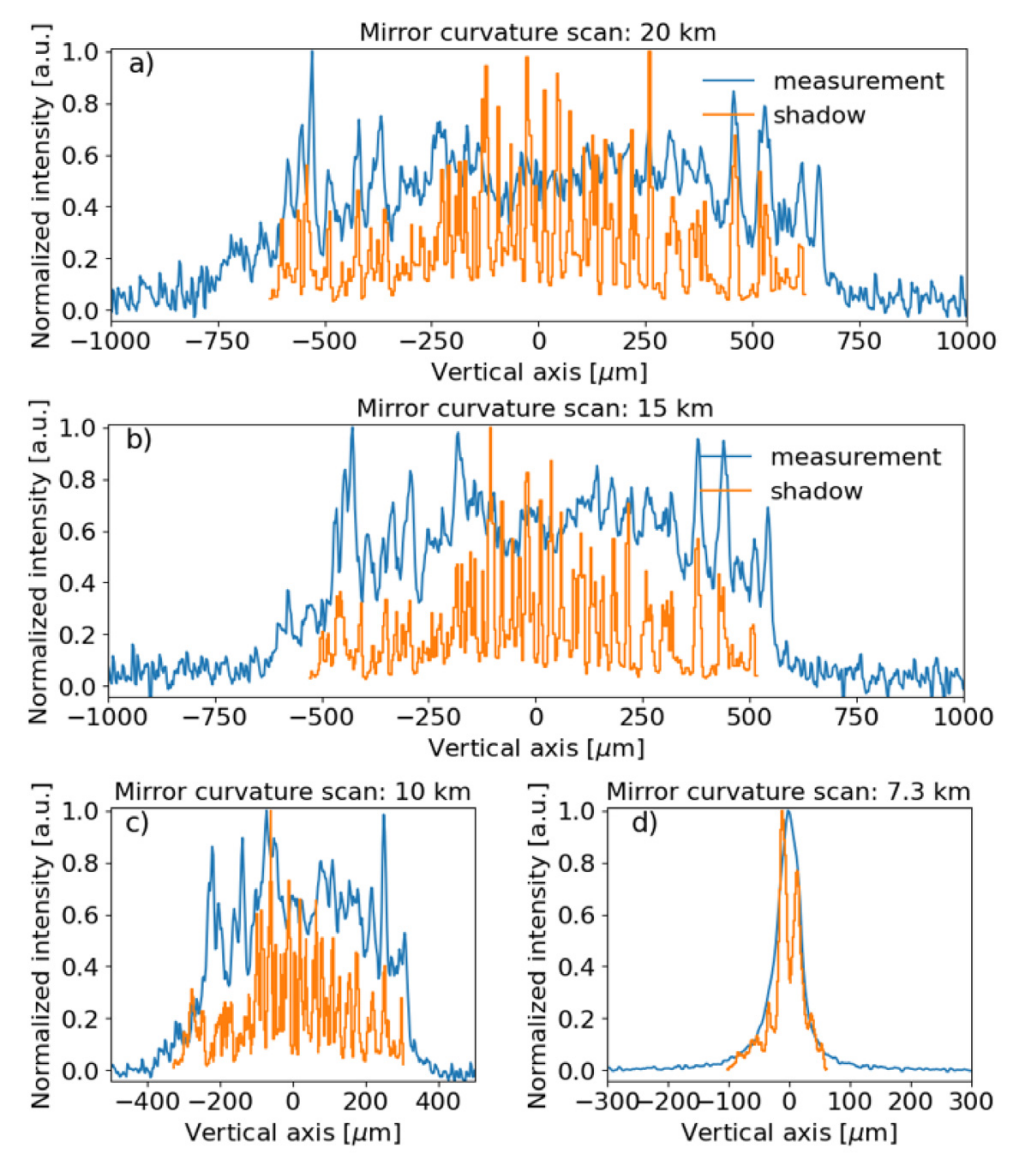
Experimental beam intensity, obtained in polychromatic mode, projected in the vertical direction compared to SHADOW simulations for different meridional curvature radii of the toroidal mirror: **a**) 20 km, **b**) 15 km, **c**) 10 km and **d**) 7.3 km (minimum measured beam size). The measured data and simulations were normalized to the peak value. A vertical offset was applied to the experimental data for better comparison. Please notice that same plot label applies for figures **c**) and **d**).

We scanned the toroidal mirror curvature radius in polychromatic mode (pink spectrum shown in Figure 5). In this run, we performed the same type of scans as the previous (Run 3) but without the crystal monochromator. Some measurement and simulation results are shown in Figure 9.

### Run 5

**Figure 10.**
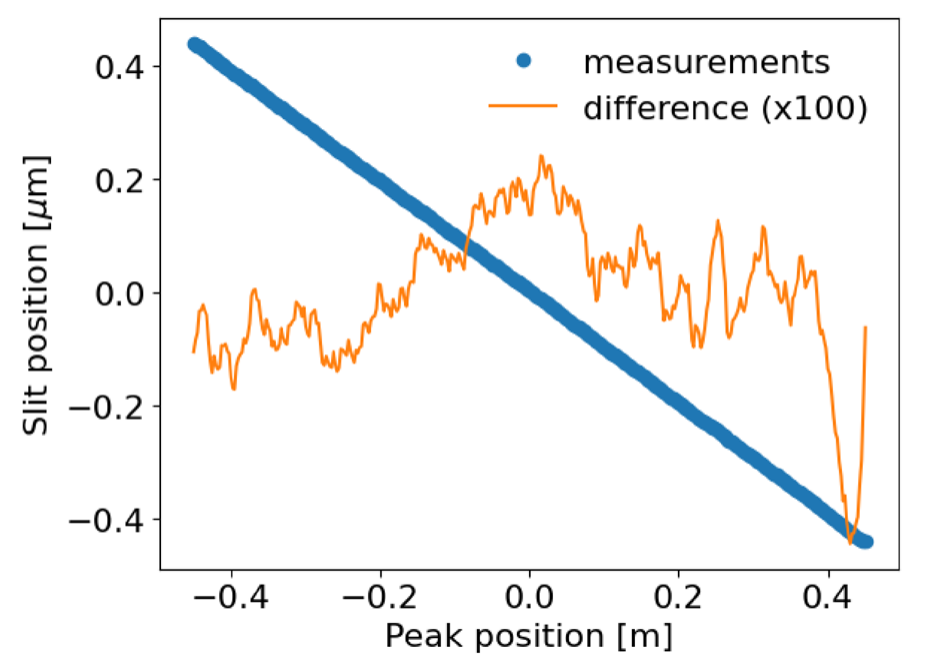
Profile Gaussian peak position as a function of the *ssh1vo* position, linear fitting and their difference. The slit scan has been centered, the difference has been multiplied by 100 for visualization purposes.

**Figure 11.**
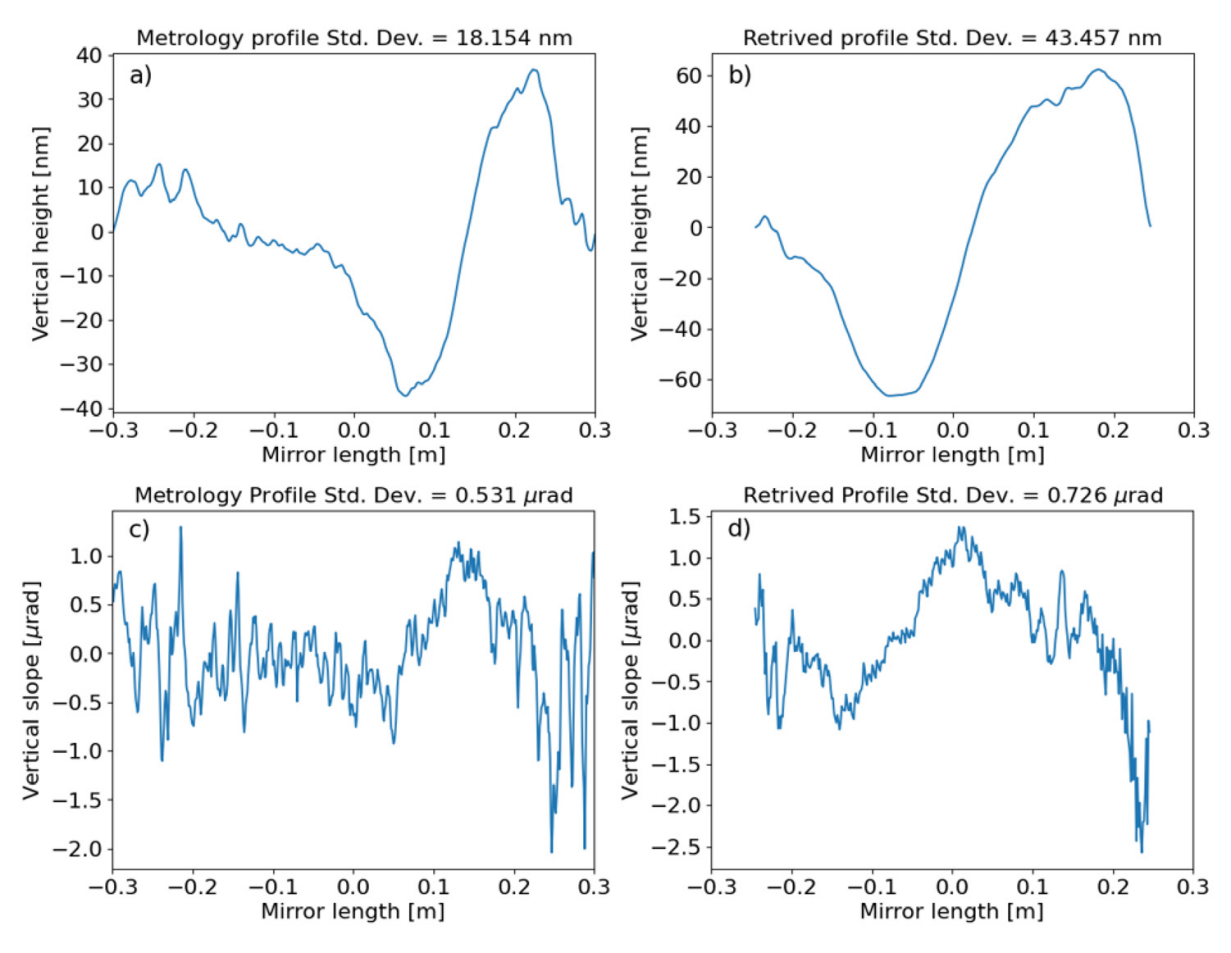
Profiles of the toroidal mirror of ID09, as measured at the European Synchrotron metrology lab (with best circular curvature detrended): **a**) Mirror heights and **c**) slopes. And retrieved by the vertical slit scan measurements: **b**) heights and **d**) slopes.

The cross section images of the photon beam were recorded while the *ss1vo* slit was scanned. There is a double objective of this series of measurements. On one side, they serve to determine the pixel size, as explained in the subsection “Calibration of pixel size”. On the other side, these measurements can be used to obtain an approximated slope profile of the mirror. If the toroidal mirror were perfect, the relation between the peak position ^4^ for each *ssh1vo* position ( Figure 2) would be perfectly linear. In reality, due to the mirror slope errors, there are some differences. We can plot the peak position as a function of the slit position, a linear fit, and the difference between data and linear fitting (see Figure 10). Finally, we can get the slopes error profile projecting the above-mentioned difference to the mirror position and then considering its angle. The height profile is obtained by integration of the slopes profile. Results obtained in this way are compared with the metrology data in Figure 11.

## Discussion

The cross section of the monochromatic beam depends on the photon energy. Indeed, the collimated central cone at the resonant energy (18.5 keV) illuminates the central zone of the toroidal mirror, and the intensity distribution at the image plane contains a single peak (see Figure 6d, Figure 7d.). When the photon beam energy is red-shifted, the central cone splits into a ring that corresponds to two peaks in the vertical profile. The separation of the peaks increases for lower photon energies. This means that a new part of the mirror is explored. Therefore, to illuminate the whole mirror is important to use a pink beam, as in Figure 9. In the intensity profiles of Figure 9a is possible to relate some peaks of the measured profile to peaks in the simulation, originated by some structures in the mirror profile ( Figure 11a). The agreement between the ray tracing and the wave optics is very good ( *cf.* Figure 7). This indicates that both models are good at estimating the effect of the errors. Ray tracing gives good results because, under our experimental conditions, the edges of the mirror do not create significant diffraction. Also, the characteristic lengths of the surface errors of the mirror do not create coherent scattering.

**Figure 12.**
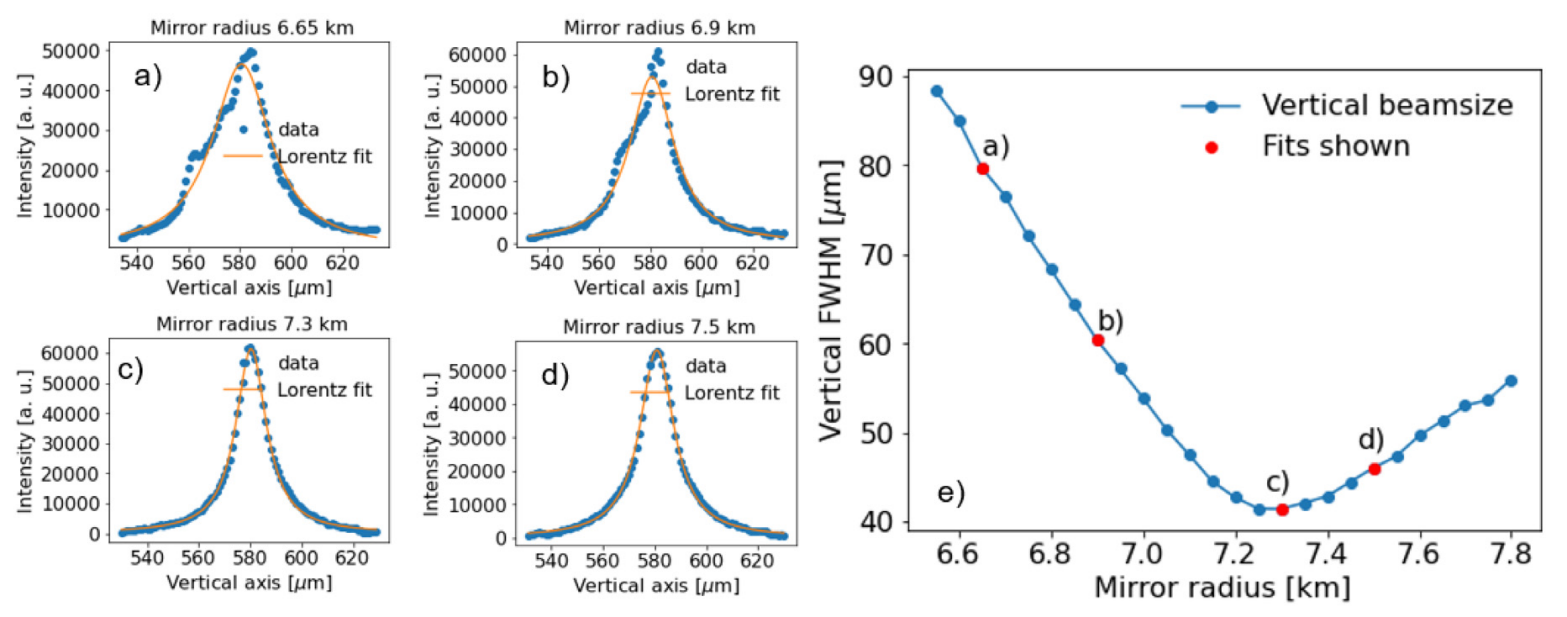
Minimum vertical size obtained from the scan of the mirror radius. The measurements correspond to the case of a monochromatic beam at the undulator resonance. The FWHM of the projected vertical profile was obtained by a Lorentz fit of the intensity profile for each radius step. For example: **a**) 6.65 km, **b**) 6.9 km, **c**) 7.3 km and **d**) 7.5 km. The results of vertical FWHM as a function of the radius are shown in **e**), where the red points correspond to the fit examples in **a**– **d**).

In general, the experimental results show broader and smoother intensity profiles than the simulated ones. The edges of the intensity profiles are sharper in the simulations. The peaks in the simulations are better defined. There are some possible reasons to explain these facts, like i) the experimental spectral distribution is wider than the calculated one ( Figure 5), and ii) the simulations used a slopes profile (metrology profiles Figure 11) limited to the clear aperture (600 mm), therefore possible illumination outside or on the edges of this aperture are not included in the simulations. The effect of the radius of curvature is analysed in Figure 8, showing a clear focusing effect when the radius is reduced from 18 km (the beam should be focused at a theoretical position 45.5 m downstream from the mirror, therefore practically unfocused at the detector plane) to 7.3 km, which correspond to the minimum vertical spot recorded at the detector plane. At 7.3 km, the experimental focused beam looks wider than the simulated ones. One reason is probably due to errors in the calibration of the radius of curvature. From the lens equation of meridional focusing,


$$\left(\frac{1}{p}+\frac{1}{q}\right)\frac{\cos\theta }{2}=\frac{1}{R},\;(1)$$

with *p* the distance between the mirror and the source, *q* the distance from the mirror to the image, *θ* the mirror incidence angle and *R* the mirror meridional radius, the best focus is expected at 6.9 km, contrary to the experimental value of 7.3 km. ( Figure 12).

**Figure 13.**
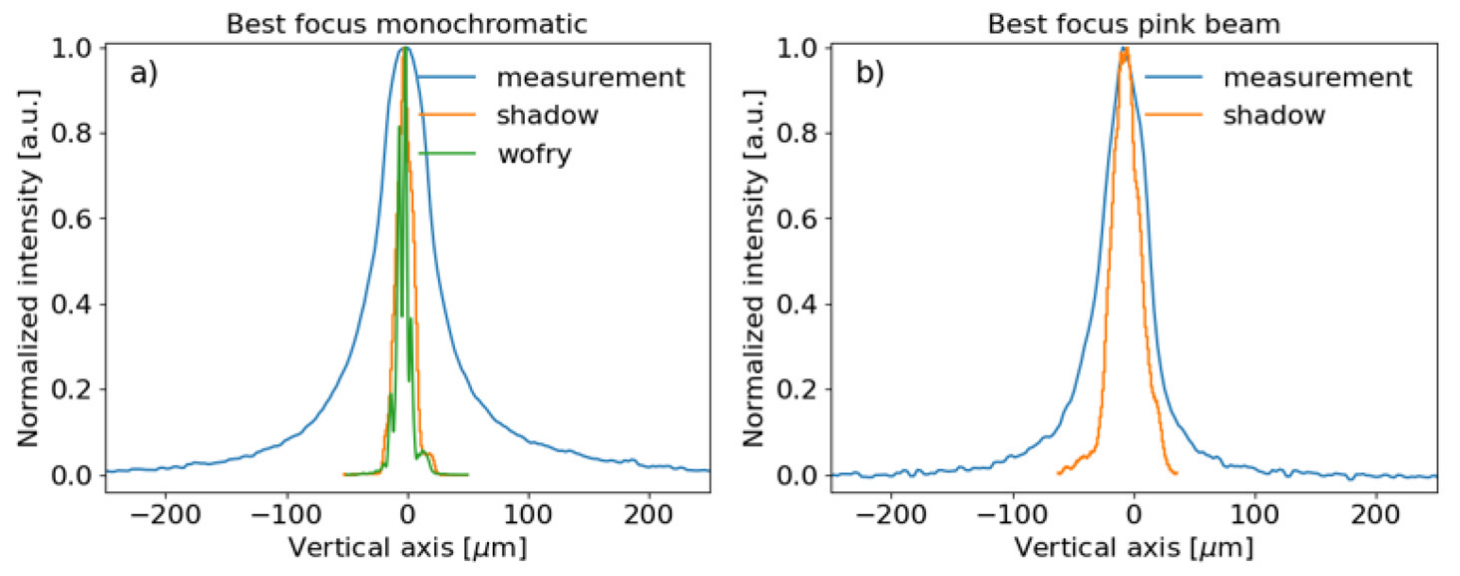
Comparison between best focusing vertical beam profiles: **a**) Monochromatic photon beam at 18.5 keV and **b**) Pink photon beam.

The best theoretical (R=6.9 km) and experimental (R=7.3 km) focusing spots are compared in Figure 13. While the experiment presents a wider peak than the simulations for the monochromatic case, the difference is considerably less in the pink beam case, therefore the influence of errors in the monochromator crystals surfaces can not be discarded.

Another possible explanation of the observed broadening in the experimental profiles could be the detector point spread function, which seems to be larger than about 10 *µ*m, as deduced from the thin lines in Figure 2– Figure 5.

**Figure 14.**
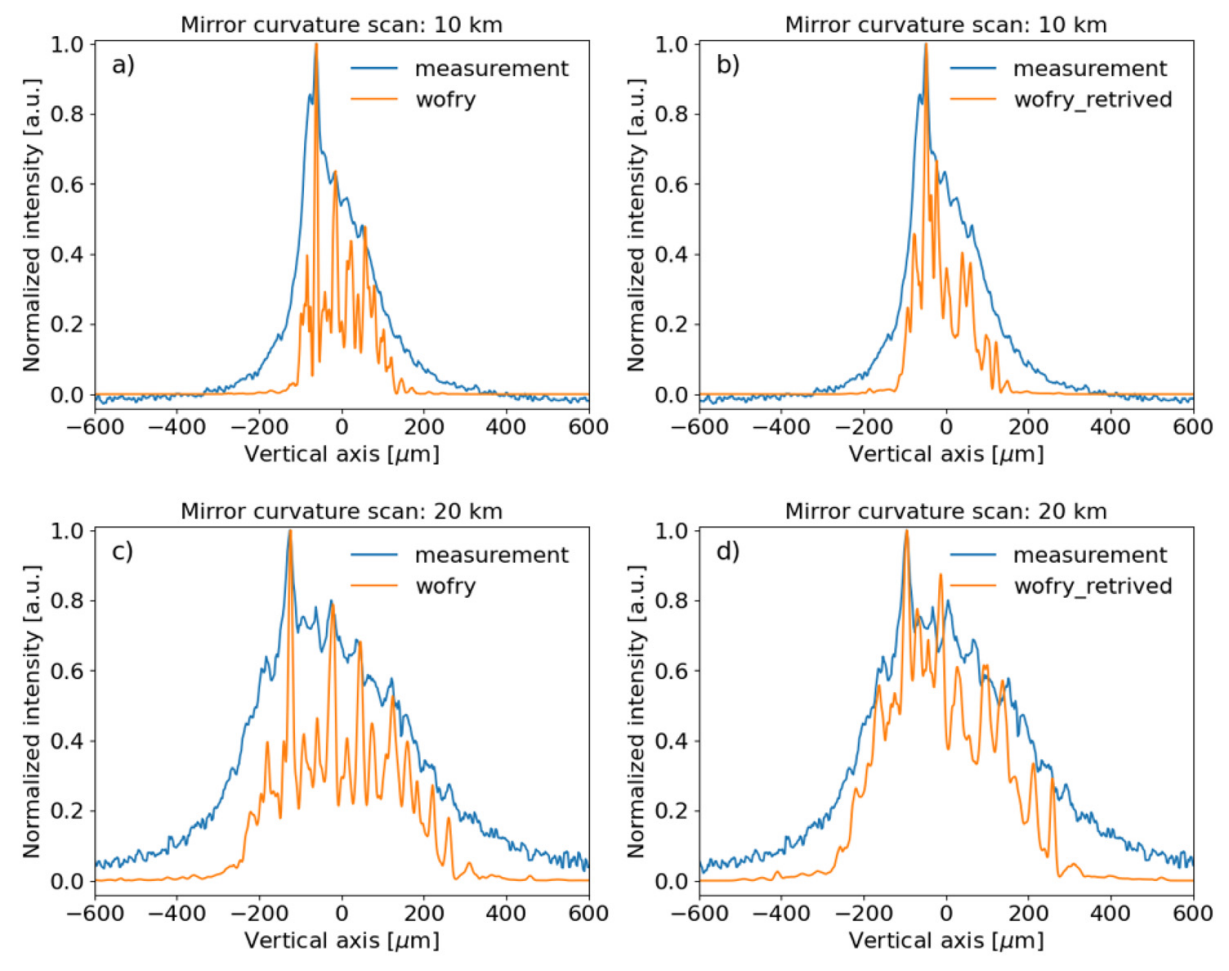
Wavefront simulations using WOFRY1D compared to measurements for a few of meridional curvature radius of the toroidal mirror. Left: using the mirror profile from metrology measurements for mirror radius 10 km ( **a**) and 20 km ( **c**). Right: using the *in-situ* retrieved profile for mirror radius 10 km ( **b**) and 20 km ( **d**) (see Figure 11 and text).

The recording of a multitude of images while scanning the slit *ss1vo* constitutes a method of *in-situ* metrology. The small slit gap allows looking at a small transversal line on the mirror surface. The position at which the intensity peak compared to the theoretical position for a zero-error mirror can be used to calculate the slope of the mirror profile. This method allowed us to record the mirror profile ( Figure 11). Although there is an agreement in the trends of the slope and height profiles, as well as in the amplitudes, there is not a quantitative agreement. This can be due to the possibility that the mirror error profile evolved from what was recorded in the metrology lab in 2017, because of its mounting in the beamline and the cycles of bending-unbending that suffered. Also, the experimental parameters (slit position and gap, detector resolution) could be improved. An optimized setup would conduct to more accurate *in-situ* metrology. We performed a few simulations using the *in-situ* recorded toroidal mirror profiles ( Figure 14). The results agree qualitatively with the experiment, again, some discrepancies exist in the resolving power of the peaks and overall peak widths. This is certainly due to the fact that the experimental beam is less ideal than the simulated beam, because of some smearing effects that need further investigation.

In general, we observed good agreement between simulations, either ray tracing (incoherent beam) or with wave optics (partially coherent simulations), with the measurements. This indicates that the effects strictly related to coherence are not very important.

## Conclusions

We have recorded images of the X-ray beam cross section after being reflected by a toroidal mirror with the aim of studying how the surface errors of the mirrors degrade the beam quality. The beam (monochromatic or pink, around 18.5 keV) is imaged out of focus. The experimental results are compared with simulations using ray tracing based on geometrical optics and partial coherence based on coherent mode decomposition and physical optics. There is a good qualitative agreement between experimental and simulated results. However, full quantitative agreement is not obtained because of different facts, like the possible evolution with time of the mirror height profile since it was measured, or other effects affecting the beam and not included in the simulations. This will be the object of further investigations.

The experimental setup was also employed to obtain a profile of the slope errors of the mirrors. Even though the distance between the *ssh1vo* slits and the *xeye* is quite short as compared with the distance from the toroidal mirror to the *xeye*, retrieved a profile that improved the simulations ( Figure 14d).

In conclusion, we learned that for our experimental conditions, the fringes observed in the unfocused beam are directly related to the low spatial frequency structures presented in the mirror profiles, and are not dependent on the coherence of the beam. Both classical ray tracing calculations and partially coherent simulations using coherent mode decomposition are validated as accurate techniques for these simulations. A simple slit scan can be efficiently used to retrieve *in-situ* the approximated mirror profile, a method that could be optimized to gain in resolution.

## Ethics and consent

Ethical approval and consent were not required.

## Data Availability

The experimental data from these measurements are available in 10. The experimental logbook, OASYS workspaces and scripts used for the analysis are 11. Zenodo: Beam profiles images measured of an undulator-produced X-ray beam reflected by a high-quality toroidal mirror https://doi.org/10.5281/zenodo.7660631 ^10^ This project contains the following underlying data, all beamprofiles for different configurations: xeye001.h5 (scanning photon beam energies) xeye002.h5 (scanning photon beam energies, corrected images) xeye003.h5 (scanning photon beam energies, horizontal primary slits = 0.1 mm) xeye004.h5 (coarse curvature radius scan of the toroidal mirror in monochromatic mode, 18.35 keV) xeye005.h5 (fine curvature radius scan of the toroidal mirror in monochromatic mode, 18.35 keV) xeye006.h5 (ss1vo vertical slit scan) xeye007.h5 (coarse curvature radius scan of the toroidal mirror in monochromatic mode, pink mode) xeye008.h5 (fine curvature radius scan of the toroidal mirror in monochromatic mode, pink mode) Data are available under the terms of the Creative Commons Attribution 4.0 International license (CC-BY 4.0). Zenodo: jureyherrera/toroidal_mirror_project: first-release. https://doi.org/10.5281/zenodo.7661335 This projects contains the following extended data: logbook (Folder containing logbook output) oasys_worflows (Folder containing Oasys workflows implemented) scripts (Folder containing supplementary Python scripts to extract and process beamprofiles from the h5 files data) Data are available under the terms of the Creative Commons Zero "No rights reserved" data waiver (CC0 1.0 Public domain dedication).
