# Supplementary material for: X-ray beam quality after a mirror reflection: Experimental and simulated results for a toroidal mirror in a 4 th generation storage-ring beamline
Source: Open Res Eur. 2023 Oct 12;3:173. [Version 1] doi: 10.12688/openreseurope.16211.1 (PMC10660299; doi:10.12688/openreseurope.16211.1)
Supplement: Peer review history

# Reviewer report: Reviewer response for version 1

**DOI:** 10.21956/openreseurope.17499.r35813

**Published:** 2023-11-20

**Authors:** Jangwoo Kim

**Affiliations:** 1 Pohang Accelerator Laboratory, Pohang University of Science and Technology, Pohang, South Korea

This interesting paper investigates the effect of beam quality by toroidal mirrors using 4th generation synchrotron radiation beams. This paper compares actual measurement data with a simulation of an ideal situation or a known error range and suggests experimental directions that will be helpful to other researchers. However, it was said that the simulation results and actual measurements were consistent, but I hope that a more quantitative analysis and evaluation will be conducted in the future. I would like to request that the authors address the minor comments listed below.

1. With advances in synchrotron radiation beams and mirror manufacturing performance, better beams and mirrors are now available. However, are there any requirements for mirror performance necessary to fully utilize the 4th generation synchrotron radiation beam with small emittance and high coherence fraction? Is the reason for using a mirror with 0.5 μrad rms slope error and 2.8 Å micro-roughness in this paper a manufacturing limitation? Or is it because the performance is sufficient to use 4th generation synchrotron radiation?
2. An additional explanation about the primary devices used in this paper is needed. For example, an explanation of *ss1vo* (or *ssh1vo*) and *xeye* (not typical instruments) shown in Figure 1 and a description of mirror bender are necessary. And what is the initial meridional radius of the mirror used?
3. How do you change the radius of curvature of the toroidal mirror? And how is the actual changed radius of curvature measured? What is the accuracy or error range of the set radius of curvature?
4. In Figure 11, it is mentioned that “best circular curvature detrended,” but what is the curvature? Is it a reasonable value? In the case of (b), since the quadratic function component has been removed, it appears that the cubic function component remains. What is the resolution of how we measured (b) in Figure 11? The measurement resolution does not seem to be high due to the limited pixel size of the detector and the very short slit-to-detector distance.

Is the study design appropriate and does the work have academic merit?

Yes

Is the work clearly and accurately presented and does it cite the current literature?

Partly

If applicable, is the statistical analysis and its interpretation appropriate?

Partly

Are all the source data underlying the results available to ensure full reproducibility?

Yes

Are the conclusions drawn adequately supported by the results?

Yes

Are sufficient details of methods and analysis provided to allow replication by others?

Yes

Reviewer Expertise:

X-ray Optics, X-ray Instrumentation, Metrology, Multilayer

I confirm that I have read this submission and believe that I have an appropriate level of expertise to confirm that it is of an acceptable scientific standard.

# Reviewer report: Reviewer response for version 1

**DOI:** 10.21956/openreseurope.17499.r35809

**Published:** 2023-11-16

**Authors:** Satoshi Matusuyama

**Affiliations:** 1 Materials Physics, Nagoya University, Nagoya, Aichi Prefecture, Japan; 2 Precision Engineering, Osaka University, Suita, Osaka Prefecture, Japan

This is an interesting paper that investigates the X-ray beam profiles after the reflection of a focusing mirror. In this study, the degradation of beam quality caused by shape error of a toroidal mirror was measured and compared with ones calculated by ray trace and partial coherent simulations. Various studies have been made, but the results are not in good agreement with the calculations (at least not as well as I expected). The reasons are honestly explained. However, I am not pessimistic about these results, since it is difficult to obtain accurate parameters of the optical devices used and the incident beams in practical cases. This study is a good example of trial-and-error, which can be published as a reference for other beamline scientists. It is interesting to note that the recalculated results at the end, based on the *in-situ* measured slope error, are in relatively good agreement. It suggests that the calculation can be done correctly if accurate information is available. I don't have any strong negative comments about the paper, but I believe the following minor comments will improve this manuscript.

 [Minor comments]

 [1] Fig. 3 and related descriptions

 I don’t understand Fig. 3. Please explain Fig. 3 in detail. The graph has no axis titles. I don't understand why you do that to know the pixel size. Usually, the camera is scanned with the slit fixed.

 [2] *in-situ* metrology

 Please cite appropriate references. This is a paper I came up with immediately ^1^.

 [3] *xeye*

 Please cite appropriate references to know the details of the camera.

 [4] page 10, “ *(cf. Figure 7). This indicates that both models are good at estimating the effect of the errors. Ray tracing gives good results because, under our experimental conditions, the edges of the mirror do not create significant diffraction.*”

 I need more explanations to understand the result. Do you mean that in this case the coherence of the incident beam is underestimated and therefore the wofly provides inaccurate beam profiles?

Is the study design appropriate and does the work have academic merit?

Yes

Is the work clearly and accurately presented and does it cite the current literature?

Partly

If applicable, is the statistical analysis and its interpretation appropriate?

Yes

Are all the source data underlying the results available to ensure full reproducibility?

Yes

Are the conclusions drawn adequately supported by the results?

Yes

Are sufficient details of methods and analysis provided to allow replication by others?

Yes

Reviewer Expertise:

X-ray optics

I confirm that I have read this submission and believe that I have an appropriate level of expertise to confirm that it is of an acceptable scientific standard.

## References

[1] : Incoherent x-ray mirror surface metrology. *Proc. SPIE 3152, Materials, Manufacturing, and Measurement for Synchrotron Radiation Mirrors* .1997; 10.1117/12.295559 10.1117/12.295559
